# Abnormal regional signal in the left cerebellum as a potential neuroimaging biomarker of sudden sensorineural hearing loss

**DOI:** 10.3389/fpsyt.2022.967391

**Published:** 2022-07-22

**Authors:** Lei Liu, Jun Fan, Hui Zhan, Junli Huang, Rui Cao, Xiaoran Xiang, Shuai Tian, Hongwei Ren, Miao Tong, Qian Li

**Affiliations:** ^1^Department of Otorhinolaryngology, Tianyou Hospital, Affiliated to Wuhan University of Science and Technology, Wuhan, China; ^2^Department of Medical Imaging, Tianyou Hospital, Affiliated to Wuhan University of Science and Technology, Wuhan, China; ^3^Department of Stomatology, Tianyou Hospital, Affiliated to Wuhan University of Science and Technology, Wuhan, China

**Keywords:** sudden sensorineural hearing loss, regional homogeneity, resting-state fMRI, support vector machine, neuroimaging biomarker

## Abstract

**Objective:**

While prior reports have characterized visible changes in neuroimaging findings in individuals suffering from sudden sensorineural hearing loss (SSNHL), the utility of regional homogeneity (ReHo) as a means of diagnosing SSNHL has yet to be established. The present study was thus conducted to assess ReHo abnormalities in SSNHL patients and to establish whether these abnormalities offer value as a diagnostic neuroimaging biomarker of SSNHL through a support vector machine (SVM) analysis approach.

**Methods:**

Resting-state functional magnetic resonance imaging (rs-fMRI) analyses of 27 SSNHL patients and 27 normal controls were conducted, with the resultant imaging data then being analyzed based on a combination of ReHo and SVM approaches.

**Results:**

Relative to normal control individuals, patients diagnosed with SSNHL exhibited significant reductions in ReHo values in the left cerebellum, bilateral inferior temporal gyrus (ITG), left superior temporal pole (STP), right parahippocampal gyrus (PHG), left posterior cingulum cortex (PCC), and right superior frontal gyrus (SFG). SVM analyses suggested that reduced ReHo values in the left cerebellum were associated with high levels of diagnostic accuracy (96.30%, 52/54), sensitivity (92.59%, 25/27), and specificity (100.00%, 27/27) when distinguishing between SSNHL patients and control individuals.

**Conclusion:**

These data suggest that SSNHL patients exhibit abnormal resting-state neurological activity, with changes in the ReHo of the left cerebellum offering value as a diagnostic neuroimaging biomarker associated with this condition.

## Introduction

Sudden sensorineural hearing loss (SSNHL) is a medical emergency wherein affected individuals present with sensorineural hearing loss (≥30 dB over ≥3 consecutive frequencies) of unknown origin within 3 days. SSNHL is often accompanied by symptoms including tinnitus, vertigo, and aural fullness (1). SSNHL is estimated to affect 5–27 per 100,000 persons, and its annual incidence continues to rise (2, 3). Just 5% of SSNHL cases are bilateral, with most patients exhibiting a unilateral loss of hearing without any side preference (1, 4). Although the most common suspected etiologies of SSNHL in adult patients include viral infection, vascular or hematologic disease, immune-mediated disease, tumors, trauma, and other causes, the precise etiology of SSNHL remains unclear (5). SSNHL can contribute to social difficulties and psychiatric disorders in certain cases (6), with SSNHL patients exhibiting a higher risk of depression, and depression patients similarly exhibiting increased odds of developing SSNHL (7). As such, a failure to rapidly diagnose and treat SSNHL can lead to permanent hearing loss and associated adverse effects on quality of life (8). A combination of medical history information and pure tone audiometry (PTA) is generally used to diagnose SSNHL. However, imageological approaches capable of diagnosing SSNHL are lacking.

Rapid advances in neuroimaging technologies in recent years have enabled the diagnostic evaluation of a range of neurological systems. For example, increases in the fractional amplitude of low-frequency fluctuation in the right precuneus and left superior frontal gyrus may offer value as a biomarker associated with first-episode major depressive disorder (MDD) incidence (9). Abnormal network homogeneity values of the right posterior cingulate cortex/precuneus have also been successfully used to differentiate between individuals diagnosed with obsessive-compulsive disorder and control individuals with respective sensitivity and specificity values of 67.50% and 76.32% (10). Notably, several imaging studies have reported functional and structural changes in certain brain functional networks in SSNHL patients during the acute phase (≤ 30 days) (6, 11–13). However, neuroimaging biomarkers that can be used to guide SSNHL patients diagnosis have not been reported to date.

Resting-state functional magnetic resonance imaging (rs-fMRI) is a sensitive, non-invasive imaging approach that can offer detailed insight regarding altered brain function and neuronal activity. Accordingly, rs-fMRI imaging is commonly used for the evaluation of tinnitus patients and individuals suffering from bilateral or unilateral sensorineural hearing loss (14–17). Regional homogeneity (ReHo) is a robust algorithmic approach that enables the quantification of the resting-state local synchronization of adjacent voxels (18, 19), thereby providing insight regarding the consistency of whole-brain neural activity patterns. Abnormal ReHo detected *via* rs-fMRI in particular brain regions may be indicative of aberrant spontaneous neural activity among and within these areas of the brain. Specifically, increased ReHo values correspond to improved neuronal synchrony, whereas reduced ReHo values indicate impaired local neuronal activity.

While the use of rs-fMRI approaches to study SSNHL is becoming increasingly common, no studies to date have utilized a combination of ReHo and support vector machine (SVM) approaches to analyze these rs-fMRI-derived data. SVM approaches rely on the use of a robust machine learning algorithm capable of analyzing data, recognizing patterns, and using the resultant insights to gauge diagnostic accuracy. By identifying the maximal margin separating a hyperplane, this SVM algorithm maintains enhanced generalizability and resists overfitting, providing optimal predictive accuracy for test data that have not yet been analyzed. By overlaying SVM-derived weight values onto the original brain space utilized for fMRI analyses, the areas of the brain that can most effectively differentiate between different groups of individuals can be identified (20). As SVM algorithms can effectively locate and differentiate patterns within a particular dataset, the interpretability of the associated model is improved (20). SVM analyses are ideally suited to high-dimensional datasets in which there are more features than there are samples, with multiple prior reports having achieved success in the use of SVM to identify brain states (20, 21), enabling the discrimination between individuals diagnosed with particular neurological disorders and healthy controls (9, 22–25). Whether ReHo abnormalities can be effectively employed to differentiate between individuals with and without SSNHL through an SVM analysis, however, remains to be assessed. As such, the present study was designed to explore ReHo in patients with SSNHL, to establish which brain regions exhibit abnormal SSNHL-related ReHo, and to employ an SVM approach to gauge the value of abnormal ReHo as a neuroimaging biomarker of SSNHL.

## Methods

### Subjects

Between August 2020 and December 2021, a total of 27 patients diagnosed with SSNHL and 27 normal controls from the Otolaryngology Head and Neck Surgery Department of Tianyou Hospital affiliated with Wuhan University of Science and Technology were enrolled in this study. Participants were eligible for enrollment if they exhibited: (a) ≥30 dB in ≥3 contiguous frequencies with an air-bone gap <10 dB, as measured *via* PTA; (b) a new-onset case of SSNHL with no prior history of this condition; (c) hearing loss of unknown origin; (d) no known neurological disease; (e) MRI and CT imaging results that excluded the presence of any space-occupying lesions within the intracranial space or internal auditory canal. SSNHL patients were excluded from this study if they (a) had any history of noise exposure, ototoxic drug use, or ear surgery; (b) were experiencing fluctuating hearing loss; (c) had a family history of neurological disease; or (d) exhibited any inflammation of the external or middle ear. All enrolled normal control subjects exhibited normal otoscopic tympanic membrane findings and their pure-tone air conduction thresholds <25 dB HL at 0.25, 0.5, 1, 2, 4, and 8 kHz. Control participants were also free of any history of neurological or otologic disease. Pure-tone thresholds at 0.25, 0.5, 1, 2, 4, and 8 kHz were recorded at the start and the end of treatment. The Institutional Review Board of Tianyou Hospital Ethics Committee approved this study, and all participants provided informed consent.

### Image acquisition

An Ingenia 3.0 T MRI scanner (Philips, Amsterdam, Netherlands) was used to analyze all study subjects in the Department of Medical Imaging. Participants were positioned with their heads being placed within a foam-filled prototype quadrature birdcage head coil designed to limit motion. During fMRI scanning, participants were directed to remain still and awake with their eyes close. Scanning parameters were: TR = 2000 ms, TE = 30 ms, flip angle = 90°, FOV = 220 × 220 mm^2^, matrix size = 64 × 64, slice gap = 0.7 mm, slice thickness =3.5 mm, slice number = 33, and pitch = 1 mm.

### Data preprocessing

MATLAB was used for the pre-processing of rs-fMRI data with the DPARSF software (26). Initially, the first five time points for each participant were excluded from analysis to reduce initial signal instability and to ensure that participants had sufficient time to adapt to the imaging. Samples were then corrected for head motion and slice time. Participants were not included in subsequent analyses if they exhibited over 2 mm of maximal displacement in any of the *x, y*, or *z* directions or over 2° of maximal rotation. Corrected imaging data were then subjected to spatial normalization based upon the standard Montreal Neurological Institute space, followed by resampling at 3 × 3 × 3 mm^3^. Images were then subjected to bandpass filtering (0.01–0.08 Hz) and linear detrending. Spurious covariates were then removed, including six head motion parameters derived from rigid body correction, signal from a region centered in the white matter, and signal derived from a ventricular seed-based region of interest.

### ReHo analysis

ReHo analyses were conducted using the DPABI software (http://rfmri.org/dpabi). Kendall coefficients of the time series consistency between individual voxels and neighboring voxels were used to construct ReHo brain maps, with smoothing then being performed using a Gaussian kernel with a full width and half height of 4 mm to minimize the impact of noise and deformation on the standardization process, thereby improving image effects, signal-to-noise ratio values, and statistical efficiency.

### Classification analysis

The MATLAB LIBSVM package was used to conduct SVM classification analyses in an effort to establish the ability of ReHo values extracted from abnormal regions of the brain to differentiate between SSNHL patients and normal control individuals. This analytical approach was performed using a leave-one-out technique.

### Statistical analyses

#### Demographic and clinical data

Statistical analyses were performed using SPSS 22.0, with demographic data being compared between groups using chi-square tests (sex) and two-sample *t*-tests (age). Hearing levels were compared *via* the Wilcoxon test. *P* < 0.05 was the threshold of significance.

#### ReHo values

The DPABI software was used to perform two-sample *t*-test analyses of ReHo graphs for the patient and normal control groups, with age and sex being treated as covariates. Brain templates were selected for overlay, and analyses were subjected to Gaussian Random Field (GRF) correction with a correction threshold of *P* < 0.01. Those brain regions exhibiting significant differences between these two groups were extracted as a template mask, with ReHo values for individual subjects then being extracted based on this template.

## Results

### Patient characteristics

In total, this study incorporated 27 patients diagnosed with unilateral SSNHL and 27 normal controls. There were no significant differences between these groups with respect to the age or sex of participants (*P* > 0.05), while there were significant differences between pre- and post-treatment hearing levels among SSNHL patients (*P* < 0.05). For further details regarding participant characteristics, see Table 1.

**Table 1:** Demographic and clinical characteristics.

	**SSNHL**	**NC**	***P*** **value**
Number (*n*)	27	27	–
Sex (***n***)			
Male	19	14	0.163
Female	8	13	
Age (year)	47.96 ± 9.37	44.04 ± 8.28	0.109
Hearing loss duration (day)	4.89 ± 5.86	–	–
PTA of affected ear (dB HL)			
Pre-treatment	72.50 ± 16.73	–	–
Post-treatment	60.15 ± 25.08	–	<0.001
PTA of unaffected ear (dB HL)			
Pre-treatment	24.54 ± 9.36	–	–
Post-treatment	23.83 ± 8.98	–	0.024

*Data presented as mean ± SD. n means number; SSNHL, sudden sensorineural hearing loss; NC, normal controls; PTA, pure tone audiometry. The p-value for the gender distribution was obtained by the Chi-square test. Age was compared using the two-sample t test; PTA was compared with the paired-sample Wilcoxon test. p < 0.05 was considered significant*.

### SSNHL-related ReHo abnormalities

**Figure 1:**
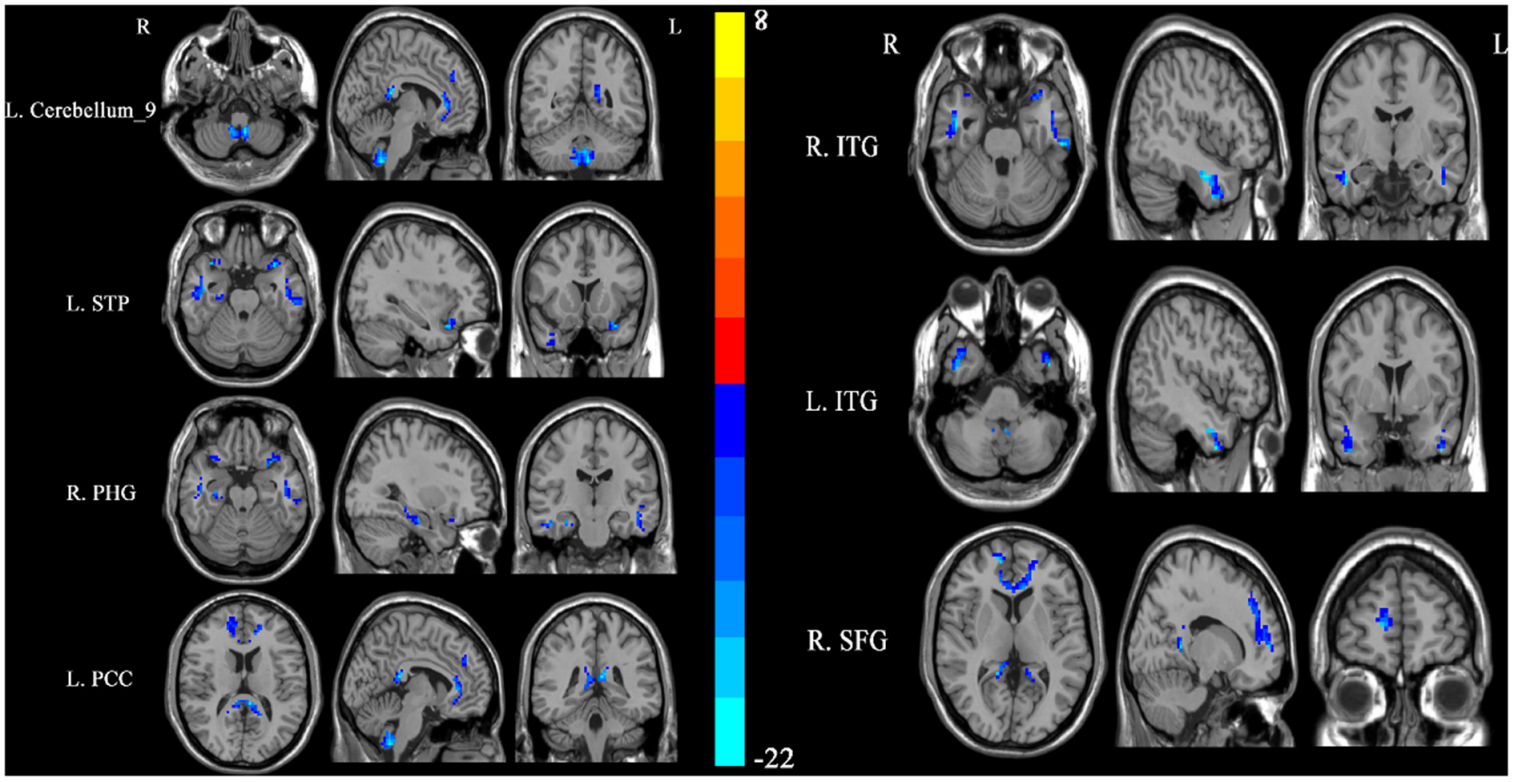
Differences in regional homogeneity (ReHo) values between SSNHL patients and normal controls. Decreased ReHo values (left cerebellum, left STP, right PHG, left PCC, right SFG, and bilateral ITG) were presented on the blue color, and the color bar indicates the T values of the group analysis. L, left; R, right; STP, superior temporal pole; PHG, parahippocampal gyrus; PCC, posterior cingulum cortex; SFG, superior frontal gyrus; ITG, inferior temporal gyrus.

SSNHL patients exhibited significant reductions in ReHo in the left cerebellum, bilateral inferior temporal gyrus (ITG), left superior temporal pole (STP), right parahippocampal gyrus (PHG), left posterior cingulum cortex (PCC), and right superior frontal gyrus (SFG) relative to normal controls (Figure 1, Table 2).

**Table 2:** Clusters with abnormal regional homogeneity in the patients with SSNHL.

**Cluster location**	**Peak (MNI)**			**Number of voxels**	***T*** **-value**
	***X***	***Y***	***Z***		
Left cerebellum	−6	−51	−54	121	−22.4941
Right ITG	45	−9	−27	142	−22.6682
Left ITG	−45	3	−42	145	−23.2774
Left STP	−33	15	−24	36	−20.5900
Right PHG	30	−18	−21	36	−17.1846
Left PCC	−6	−39	15	128	−21.3446
Right SFG	15	57	6	410	−21.1893

*SSNHL, sudden sensorineural hearing loss; MNI, Montreal Neurological Institute; ITG, inferior temporal gyrus; STP, superior temporal pole; PHG, parahippocampal gyrus; PCC, posterior cingulum cortex; SFG, superior frontal gyrus*.

### SVM analysis results

**Figure 2:**
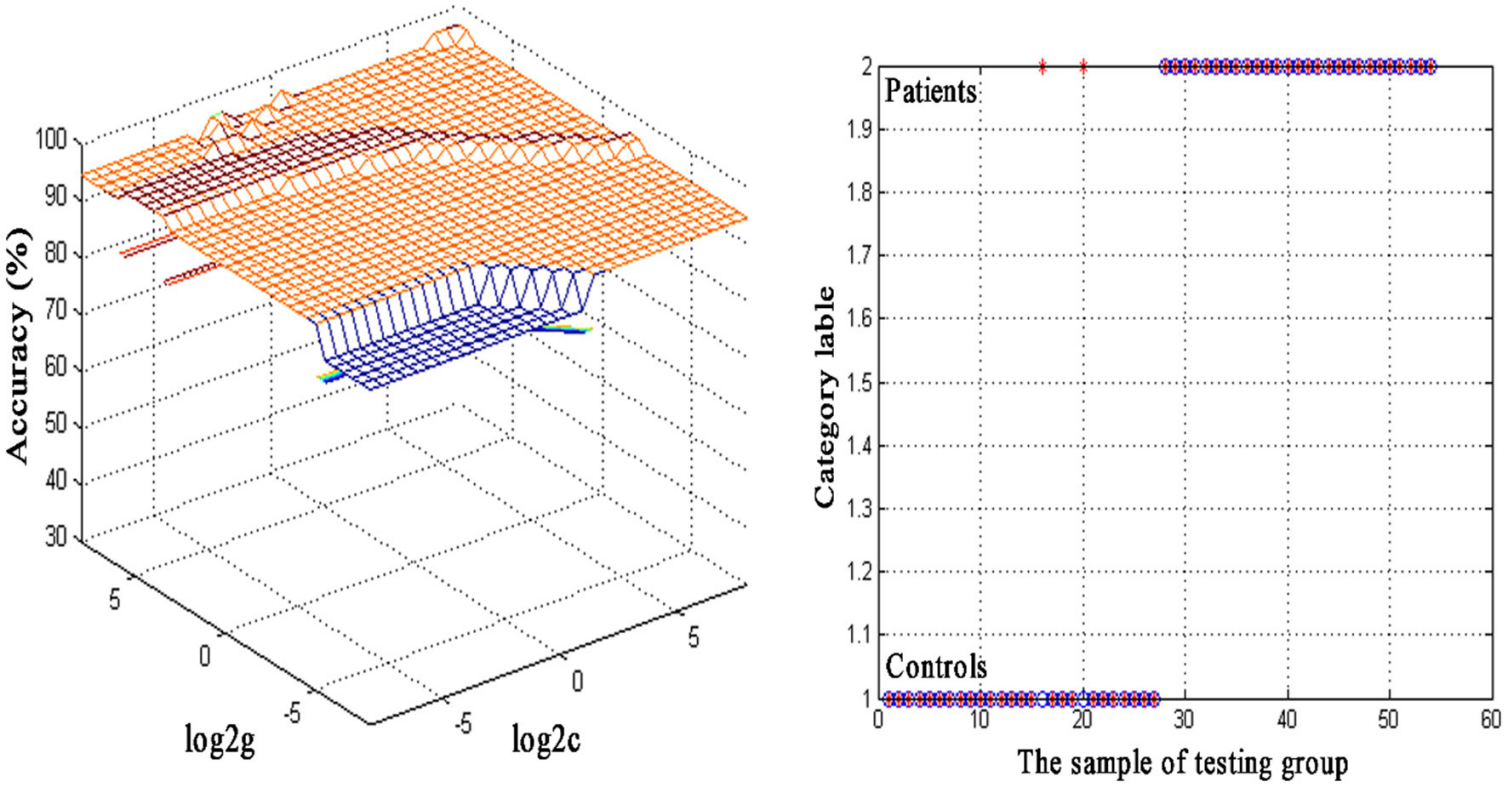
Visualization of classifications through support vector machine (SVM) using the decreased regional homogeneity (ReHo) values in the left cerebellum to discriminate SSNHL patients from normal controls. Left: SVM parameters result of 3D view. g means gamma, c means penalty coefficient. Right: Classified map of the ReHo values in the left cerebellum. Blue circle means true value and the red asterisk means predict value.

An SVM approach was used to separately analyze abnormal ReHo values in seven regions of the brain (left cerebellum, bilateral ITG, left STP, right PHG, left PCC, and right SFG), revealing decreased ReHo in the left cerebellum to exhibit the highest diagnostic accuracy (96.30%, 52/54) when differentiating between SSNHL patients and normal controls, with a sensitivity of 92.59% (25/27) and a specificity of 100.00% (27/27) (Figure 2).

## Discussion

Here, whole-brain ReHo was assessed at rest in both SSNHL patients and normal control individuals. Significant reductions in ReHo values were observed in the left cerebellum, bilateral ITG, left STP, right PHG, left PCC, and right SFG of SSNHL patients during the acute hearing loss period relative to control individuals. These data suggest that these seven regions of the brain exhibit abnormal spontaneous neural activity in individuals affected by acute-phase SSNHL. SVM analyses further indicated that reductions in ReHo in the left cerebellum may offer value as a neuroimaging biomarker that can distinguish between patients with SSNHL and unaffected controls.

The reduced ReHo of seven brain regions in SSNHL may present the baseline abnormality of sensory cortices in SSNHL at rest. Recent work suggests that the cerebellum plays a central role in the coordination of emotional, sensory, and cognitive processes (27, 28). Moreover, the cerebellum mediates the processing of acoustic information derived from auditory-associated brain regions (29). Xu et al. (15) determined that patients suffering from long-term moderately severe bilateral sensorineural hearing loss exhibit atypical patterns of spontaneous neural activity within the cerebellum, in line with the results of this present study. Here, decreased ReHo of the left cerebellum may reflect abnormal brain function in sensory and cognitive information processing in SSNHL patients, and an adaptation to engage other sensory systems as a compensatory mechanism for the acute hearing impairment. Thus, we speculated that the lack of sufficient acoustic input may have impaired the function of the cerebellum as reflected by reduced ReHo values in this brain region. The SVM analysis results for this region further yielded an accuracy of 96.30% (52/54), suggesting that reductions in left cerebellar ReHo values may offer utility as a putative neuroimaging biomarker that can aid in diagnosing SSNHL.

The ITG has been found to play a role in multiple functional brain networks associated with emotional regulation, language comprehension, memory, and visual processing (30–32). Much like the ITG, the temporal pole is a component of the association complex associated with emotion, language processing, and the multimodal integration of sensory inputs (33–37), and both the STP and ITG are components of the temporal lobe. Therefore, abnormal activity of the two brain regions could influence the function of the temporal lobe. The temporal lobe is the location of the auditory center, and it is also involved in functions relating to emotion, speech, balance, memory, and visual perception. Thus, SSNHL could lead to structural and functional impairment of the temporal lobe. The PHG is a center that is reportedly associated with higher-order cognitive functions such as visual-spatial processing and the encoding/retrieval of memories (38, 39). Lin et al. (38) conducted a tractography analysis that confirmed the existence of extensive cortico-cortical correlations between the PHG and other regions of the brain including the occipital, parietal, temporal, and frontal cortices. The temporal pole has also been regarded as a component of the parahippocampal region in humans as well as in non-human primates (39, 40). As such, the ITG, STP, and PHG are closely related to one another both functionally and structurally. Multiple reports have identified abnormal changes in the PHG and ITG in individuals suffering from hearing loss. Yang et al. (41) determined that individuals affected by long-term right-sided unilateral hearing loss exhibit significantly lower gray matter volumes in the right PHG, left superior/middle/inferior temporal gyrus, bilateral PCC, and precuneus. Chen et al. (42) also observed lower ReHo values in the precuneus, PHG, superior temporal gyrus, and inferior parietal lobe in presbycusis patients. In the present analysis, SSNHL patients exhibited resting-state ReHo abnormalities in the STP, ITG, and PHG, thus suggesting a role for these regions in the pathophysiology of this condition.

The PCC is a component of the posteromedial cortex located in the medial portion of the inferior parietal lobe (43). As a key default mode network (DMN) component, the function of the PCC has been linked to cognition, emotional regulation, and the retrieval of episodic memories. The DMN consists of the medial prefrontal cortex, precuneus, inferior parietal lobe, and PCC (44), and plays roles in negative ruminations, memory retrieval, cognitive functioning, and self-related thoughts (44–46). Reductions in ReHo values in the PCC in the present analysis are likely reflective of altered spontaneous neural activity in this region and between the PCC and connected regions. The SFG, located in the superior prefrontal cortex, has previously been separated based on diffusion tensor tractography into the anteromedial, dorsolateral, and posterior SFG subregions by Li et al. (47), revealing both the anteromedial and dorsolateral SFG to be primarily connected with the DMN when conducting resting-state analyses of functional connectivity. The SFG is also an integral component of emotional processing, cognitive control, and working memory (48–51), and several reports have documented altered ReHo values in the SFG. Patients with MDD, for example, reportedly exhibit elevated ReHo values in the left SFG, medial superior frontal gyrus, and left middle temporal gyrus (52). Relative to healthy individuals, diabetic optic neuropathy patients also exhibit significant reductions in ReHo values in the SFG, left anterior cingulate, and right middle frontal gyrus (53). Diabetic vitreous hemorrhage patients also reportedly present with significant increases in bilateral SFG, bilateral cerebellar posterior lobes, and right superior/middle occipital gyrus ReHo values (54). However, few studies to date have explored SSNHL-related changes in ReHo values in the SFG. Here, SSNHL patients were found to exhibit significantly reduced ReHo values in the right SFG as compared to normal controls, consistent with the weakening of the consistency of spontaneous neural activity in this region. The observed ReHo abnormalities in the PCC and SFG may thus be suggestive of abnormal structures in the DMN in patients affected by SSNHL.

Prior research has revealed SSNHL incidence to be correlated with higher rates of affective disorders including depression and anxiety (55, 56). The results of this analysis suggest that acute auditory deprivation may alter spontaneous neuronal activity following SSNHL incidence, contributing to changes in higher-order brain functions. Together, these results may offer new insight regarding the underlying neuropathological mechanisms of SSNHL and associated alterations in higher-order brain functions.

The advent of novel imaging technologies has spurred a growing interest in the use of neuroimaging biomarkers as tools for the diagnosis and monitoring of a range of diseases and disorders. Gao et al. (25) reported that the increased degree centrality of the right inferior parietal lobule and the left dorsolateral superior frontal gyrus could be used as a combined imaging biomarker of right temporal lobe epilepsy (rTLE) with respective sensitivity and specificity values of 100% and 98.55%. Abnormal ReHo values in particular brain regions have also shown promise as a tool for differentiating between schizophrenia patients and healthy controls (57). This study, however, is the first to have employed an SVM approach to examine the utility of ReHo abnormalities as SSNHL-related neuroimaging biomarkers. ReHo is an indicator of local brain regions activity. SVM is a mature and effective algorithm in machine learning and it has successfully been used in prior reports to guide the diagnosis of first-episode MDD (9), schizophrenia (58), obsessive-compulsive disorder (10), and rTLE (25). Here, an SVM approach was used to analyze the diagnostic utility of abnormal ReHo values in the left cerebellum, bilateral ITG, left STP, right PHG, left PCC, and right SFG, revealing that the left cerebellum exhibited respective accuracy, sensitivity, and specificity values of 96.30%, 92.59%, and 100.00% when differentiating between SSNHL patients and normal controls.

There are certain limitations to this analysis. For one, the sample size was relatively limited. Additionally, scanner-derived noise could not be fully eliminated even though participants were provided with earplugs. Lastly, no analyses of the correlations between ReHo and clinical findings were performed.

## Conclusion

In conclusion, these results suggest that reduced ReHo in the left cerebellum, bilateral ITG, left STP, right PHG, left PCC, and right SFG correspond to abnormal spontaneous brain activity in patients diagnosed with SSNHL. Moreover, decreases in ReHo within the left cerebellum may offer value as a neuroimaging biomarker that can aid in the diagnosis of SSNHL.

## Data availability statement

The original contributions presented in the study are included in the article/supplementary material, further inquiries can be directed to the corresponding author.

## Ethics statement

The studies involving human participants were reviewed and approved by the Institutional Review Board of Tianyou Hospital Ethics Committee. The patients/participants provided their written informed consent to participate in this study.

## Author contributions

LL, JF, and HZ designed the experiment and wrote this article. JH, RC, XX, ST, and HR collected and analyzed these data. MT and QL guided the experiment and revised the article. All authors contributed to the article and approved the submitted version.

## Funding

This research was supported by the grant from the Education Department of Hubei Province scientific research project (Grant No. D20201101).

## Conflict of interest

The authors declare that the research was conducted in the absence of any commercial or financial relationships that could be construed as a potential conflict of interest.

## Publisher's note

All claims expressed in this article are solely those of the authors and do not necessarily represent those of their affiliated organizations, or those of the publisher, the editors and the reviewers. Any product that may be evaluated in this article, or claim that may be made by its manufacturer, is not guaranteed or endorsed by the publisher.
